# ﻿Taxonomic review of the genus *Ponyalis* Fairmaire, 1899 (Coleoptera, Lycidae), with descriptions of six new species from China

**DOI:** 10.3897/zookeys.1203.120166

**Published:** 2024-05-31

**Authors:** Chen Fang, Yuxia Yang, Xingke Yang, Haoyu Liu

**Affiliations:** 1 Key Laboratory of Zoological Systematics and Application, School of Life Sciences, Hebei University, Baoding 071002, China; 2 Hebei Basic Science Center for Biotic Interaction, Hebei University, Baoding 071002, China; 3 Key Laboratory of Zoological Systematics and Evolution, Institute of Zoology, Chinese Academy of Sciences, Beijing 100101, China

**Keywords:** Aedeagus, alpha taxonomy, antennae, differential diagnosis, distribution, identification key, Net-winged beetles, Oriental Region

## Abstract

The lycid genus *Ponyalis* Fairmaire, 1899 is reviewed. Six new species are described from China, including *P.longicornis***sp. nov.**, *P.truncata***sp. nov.**, *P.dabieshanensis***sp. nov.**, *P.hainanensis***sp. nov.**, *P.quadricollimima***sp. nov.**, and *P.zhejiangensis***sp. nov.** Nine previously known species, including *P.alternata* (Pic, 1927), *P.fukiensis* (Bocak, 1999), *P.gracilis* (Bocak, 1999), *P.himalejica* (Bourgeois, 1885), *P.klapperichi* (Bocak, 1999), *P.laticornis* Fairmaire, 1899, *P.nigrohumeralis* (Pic, 1939), *P.quadricollis* (Kiesenwetter, 1874), and *P.variabilis* Li, Bocak & Pang, 2015 are illustrated with images of the habitus and aedeagi to make the comparisons with the new species. In addition, a distribution map and an identification key to all 24 species of *Ponyalis* are provided.

## ﻿Introduction

The genus *Ponyalis* Fairmaire, 1899 is currently classified in the lycid tribe Lyponiini (Bocak and Bocakova 1990, 2008; Kazantsev 2002, 2005; Kusy et al. 2019), which includes one other genus, *Lyponia* Waterhouse, 1878. These two genera have been confused for a long time due to their morphological similarities (e. g., Gorham 1883; Kleine 1924; Pic 1926; Nakane 1961, 1969). It was not until Bocak (1999) conducted a cladistics analysis based on morphological data, where he identified *Ponyalis* as a subgenus of *Lyponia*. Later, Kazantsev (2002) reinstated *Ponyalis* as a separate genus because of its morphological differences from *Lyponia*, which are as follows: basal part of the coxite free, while basally fused in *Lyponia*; antennomere I abruptly widened near base in both sexes, while progressively widened towards apex in *Lyponia*; elytral primary costa III almost reaching apex, while not extending beyond apical fifth in *Lyponia*; aedeagus present with a pair of lateral thorns in the preapical portion of the median lobe, while absent in *Lyponia* (Kazantsev 2002). Kazantsev’s (2002) classification system was adopted by Li et al. (2015a) and supported by their molecular phylogenetic results of Lyponiini (Li et al. 2015b).

Prior to this study, a total of 18 *Ponyalis* species are described, which are widely distributed in the eastern Palaearctic and Oriental regions (Bocak 1999; Bocakova and Bocak 2007; Li et al. 2015a). During our study, we assembled a large series of *Ponyalis* material, which made it possible for us to review this genus. After examination and identification, we discovered six new species from China, which are reported in the present study. Meanwhile, some of the previously known species are illustrated in more detail to make them better known, and enable comparison with the new species, allowing a better understanding of the species diversity of the Chinese *Ponyalis* fauna.

## ﻿Materials and methods

The studied specimens are deposited in the Institute of Zoology, Chinese Academy of Sciences, Beijing, China (**IZAS**), Entomological Museum of China Agricultural University, Beijing, China (**CAU**) and Museum of Hebei University, Baoding, China (**MHBU**).

Studied specimens were first softened in water, and then the genitalia of both sexes were dissected. After dissection, the male genitalia was cleared in a 10% NaOH solution, examined and photographed in glycerol, and finally glued on a paper card for permanent preservation. Images of the adults were taken with a Canon EOS 80D digital camera, and those of the genitalia by a Leica M205A stereomicroscope, which were stacked in Helicon Focus 7. The final plates were edited in Adobe Photoshop CS3.10.0.1.

The measurements were taken with Image J 1.50i (NIH, Bethesda, MD, USA). Body length was measured from the anterior margin of the head to the elytral apex, and the width across the elytral humeri. Pronotal length was measured from the middle of the anterior margin to the middle of posterior margin of the pronotum and the width across its widest part. Eye diameter was measured at the maximal width and the interocular distance at the minimal point. The shapes of male antennomeres were assessed based on the ratio of the apical process to corresponding stem of the antennomere in length. We considered the antennomere as triangular if the ratio was at most 1.0, otherwise as lamellate if a higher value.

The distribution information was collected from the original publications (Bocak 1999; Kazantsev 2002; Li et al. 2015a) and the newly collected material. The distribution map was prepared by the ArcMap 10.8 and edited in Adobe Photoshop CS3.10.0.1.

## ﻿Taxonomy

### ﻿Genus Ponyalis

Taxon classificationAnimaliaColeopteraLycidae

Fairmaire, 1899

D026BC6F-7113-5656-8A8D-088C8CB5EC5B

#### Diagnosis.

Body length 9.5–15.0 mm, brown to black, pronotum red or black, elytra uniformly red but sometimes black at margins. Head small, hemispherical eyes prominent. Antennae flabellate in males, while serrate in females; antennomere I abruptly widened near base and nearly globular or flattened dorsally, II very short, III nearly triangular, IV–X triangular to lamellate, IX slender. Pronotum subquadrate, with all margins almost straight. Elytra flat to weakly convex, subparallel-sided, usually wider in female than male; each elytron with four primary and five secondary longitudinal and many transverse costae, elytral cells mostly squared, sometimes strongly transverse. Male genitalia robust, phallus long and present with a pair of lateral thorns apically, more or less projected distad at apical margin, internal sac usually invaginated, with only apex exposed, which is a slender or thorn-shaped tube, sometimes hardly visible.

#### Included species.

*P.alternata* (Pic, 1927), *P.cincinnatus* Kazantsev, 2002, *P.chifengleei* Kazantsev, 2002, *P.daucinus* Kazantsev, 2002, *P.dolosa* (Kleine, 1924), *P.fukiensis* (Bocak, 1999), *P.gestroi* Pic, 1912, *P.gracilis* (Bocak, 1999), *P.himalejica* (Bourgeois, 1885), *P.ishigakiana* (Nakane, 1961), *P.klapperichi* (Bocak, 1999), *P.laticornis* Fairmaire, 1899, *P.nigrohumeralis* (Pic, 1938), *P.oshimana* (Nakane, 1961), *P.quadricollis* (Kiesenwetter, 1874), *P.sichuanensis* (Bocak, 1999), *P.tryznai* (Bocak, 1999), *P.variabilis* Li, Pang & Bocak, 2015., *P.longicornis* sp. nov., *P.truncata* sp. nov., *P.dabieshanensis* sp. nov., *P.hainanensis* sp. nov., *P.quadricollimima* sp. nov., *P.zhejiangensis* sp. nov.

#### Distribution

**Figure 1.**
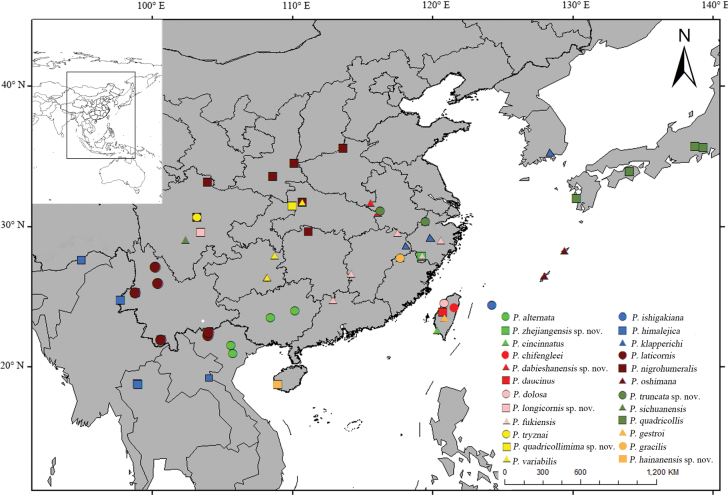
Distribution map of *Ponyalis* in the world.

**(Fig. 1**). China (Henan, Shaanxi, Gansu, Anhui, Zhejiang, Hunan, Jiangxi, Hubei, Fujian, Guangdong, Hainan, Guangxi, Chongqing, Sichuan, Guizhou, Yunnan, Xizang), Korea, Japan, Vietnam, Myanmar, Laos, Thailand, India.

##### ﻿Key to the males of *Ponyalis*

**Table:** 

1	Male antennomere IV present with a long lamella, which is ≥ 2.0× longer than joint itself and extending from middle of the joint (e.g., Figs 2C, 8A, C)	**2**
–	Male antennomere IV triangular, or present with a short lamella, which is no longer than joint itself and extending from apex of the joint (e.g., Figs 2A, 4A)	**6**
2	Elytra orange	**3**
–	Elytra red (e.g., Fig. 2C)	**4**
3	Pronotum bicolored, with a pale brown patch in center of disc; antennal tubercles pale brown; China (Taiwan) (Fig. 1)	***P.dolosa* (Kleine, 1924)**
–	Pronotum uniformly orange; antennal tubercles red; China (Taiwan) (Fig. 1)	***P.daucinus* Kazantsev, 2002**
4	Phallus strongly projected distad at apical margin (e.g., Figs 3D, E, 9A, B)	**5**
–	Phallus nearly straight at apical margin (Fig. 9D, E); China (Anhui) (Fig. 1)	***P.truncata* sp. nov.**
5	Male antennomere III with outer apical angle barely protruding laterally (Fig. 8A); phallus arched at apex in dorsal and ventral views (Fig. 9A, B); China (Anhui) (Fig. 1)	***P.dabieshanensis* sp. nov.**
–	Male antennomere III with outer apical angle strongly protruding laterally (Fig. 2C); phallus narrowly rounded at apex in dorsal and ventral views (Fig. 3D, E); China (Zhejiang, Jiangxi, Fujian, Guangdong) (Fig. 1)	***P.fukiensis* (Bocak, 1999)**
6	Male antennomere I nearly globular, elytral cells mostly squared (e.g., Fig. 10A, B)	**7**
–	Male antennomere I flattened dorsally, elytral cells transverse (e.g., Figs 6A, 7A)	**9**
7	Pronotum uniformly black; phallus strongly projected distad at apical margin and narrowly triangular at apex in ventral view (Bocak 1999: fig. 76); China (Sichuan) (Fig. 1)	***P.tryznai* (Bocak, 1999)**
–	Pronotum bicolored, black with bright red margins; phallus barely projected distad at apical margin and widely triangular at apex in ventral view (e.g., Fig. 11B)	**8**
8	Lamella of male antennomere IX 2.5× as long as joint itself (Fig. 10C); China (Sichuan) (Fig. 1)	***P.longicornis* sp. nov.**
–	Lamella of male antennomere IX 1.5× as long as joint itself; China (Sichuan) (Fig. 1)	***P.sichuanensis* (Bocak, 1999)**
9	Pronotum uniformly red or bicolored and at least bright red at margins (e.g., Fig. 7C, D)	**19**
–	Pronotum uniformly black (e.g., Figs 2A, B, 7A, B)	**10**
10	Elytra bicolored, at least black at humeri (e.g., Fig. 7A, B)	**11**
–	Elytra uniformly orange red or brownish red (e.g., Fig. 2A, B)	**12**
11	Phallus moderately widened at middle part in ventral view (Fig. 5G), weakly bent dorsally in lateral view (Fig. 5I); China (Henan, Shaanxi, Gansu, Hunan, Hubei, Sichuan) (Fig. 1)	***P.nigrohumeralis* (Pic, 1938)**
–	Phallus strongly widened at middle part in ventral view (Kazantsev, 2002: fig. 19), obviously bent dorsally in lateral view (Kazantsev, 2002: fig. 20); China (Taiwan) (Fig. 1)	***P.chifengleei* Kazantsev, 2002**
12	Lamella of male antennomere VI longer, ≥ 1.8× longer than joint itself (e.g., Fig. 6A)	**13**
–	Lamella of male antennomere VI shorter, ≤ 1.3× longer than joint itself (e.g., Fig. 2A)	**18**
13	Elytra orange-red, primary costae as strong as secondary ones	**14**
–	Elytra red to brownish red, primary costae much stouter than secondary ones (e.g., Fig. 6A, B)	**15**
14	Antennal tubercles with reddish spots posteriorly; phallus strongly widened at basal part and acute at apex (Kazantsev 2002: figs 25, 27); China (Taiwan) (Fig. 1)	***P.cincinnatus* Kazantsev, 2002**
–	Antennal tubercles uniformly black; phallus barely widened at basal part and arched at apex (Bocak 1999: fig. 75); China (Taiwan) (Fig. 1)	***P.gestroi* Pic, 1912**
15	Phallus projected distad at apical margin (e.g., Figs 9G, H, J, K, 11G, H)	**16**
–	Phallus nearly straight at apical margin (Fig. 5A, B); China (Zhejiang, Jiangxi, Fujian), Korea (Fig. 1)	***P.klapperichi* Bocak, 1999**
16	Phallus narrowly rounded at apex (Fig. 11G, H); China (Hainan) (Fig. 1)	***P.hainanensis* sp. nov.**
–	Phallus arched at apex (e.g., Fig. 9G, H)	**17**
17	Phallus hardly widened at basal part in dorsal and ventral views (Fig. 9J, K); China (Chongqing) (Fig. 1)	***P.quadricollimima* sp. nov.**
–	Phallus moderately widened at middle part in dorsal and ventral views (Fig. 9G, H); Japan (Fig. 1)	***P.quadricollis* (Kiesenwetter, 1874)**
18	Lamella of male antennomere X slender and even in width, trunk of VIII 2.8× longer than width in the middle; Japan (Fig. 1)	***P.oshimana* (Nakane, 1961)**
–	Lamella of male antennomere X broader and tapering distad, trunk of VIII 1.9× longer than width in the middle (Fig. 2A); China (Guangxi), Vietnam (Fig. 1)	***P.alternata* (Pic, 1927)**
19	Elytra ≥ 5.5× longer than pronotum (e.g., Figs 4D, 12A)	**20**
–	Elytra ≤ 5.0× longer than pronotum (e.g., Fig. 4A–C)	**21**
20	Anterior margin of pronotum straight (Fig. 4D); phallus bisinuate at lateral margins in dorsal and ventral views (Fig. 3J, K); China (Hunan, Fujian) (Fig. 1)	***P.gracilis* (Bocak, 1999)**
–	Anterior margin of pronotum arched (Fig. 12A); phallus arcuate at lateral margins in dorsal and ventral views (Fig. 11D, E); China (Zhejiang) (Fig. 1)	***P.zhejiangensis* sp. nov.**
21	Elytral primary costae much stouter than secondary ones in whole length (e.g., Fig. 7C, D)	**22**
–	Elytral primary costae nearly as strong as secondary ones (e.g., Figs 4A–C, 6C, D)	**23**
22	Phallus with > 45° angle at apex (Bocak 1999: fig. 74); Japan (Fig. 1)	***P.ishigakiana* (Nakane, 1961)**
–	Phallus with < 30° angle at apex (Fig. 5J, K); China (Hunan, Hubei, Guizhou) (Fig. 1)	***P.variabilis* Li, Bocak & Pang, 2015**
23	Pronotum present with a large black patch, extending to anterior and posterior margins; elytral width at humeri 1.5× wider than posterior margin of pronotum (Fig. 6C, D); China (Yunnan), Vietnam, Myanmar (Fig. 1)	***P.laticornis* Fairmaire, 1899**
–	Pronotum unicolored, or present with a dark brown to black patch in center of disc, but never extending to anterior or posterior margin; elytral width at humeri 1.2–1.3× wider than posterior margin of pronotum (Fig. 4A–C); China (Yunnan), Vietnam, Myanmar, Laos, Thailand, India (Fig. 1)	***P.himalejica* (Bourgeois, 1885)**

### ﻿ Ponyalis alternata

Taxon classificationAnimaliaColeopteraLycidae

(Pic, 1927)

F25CC005-AF81-5BF0-9149-07599AE046B8

**Figure 2.**
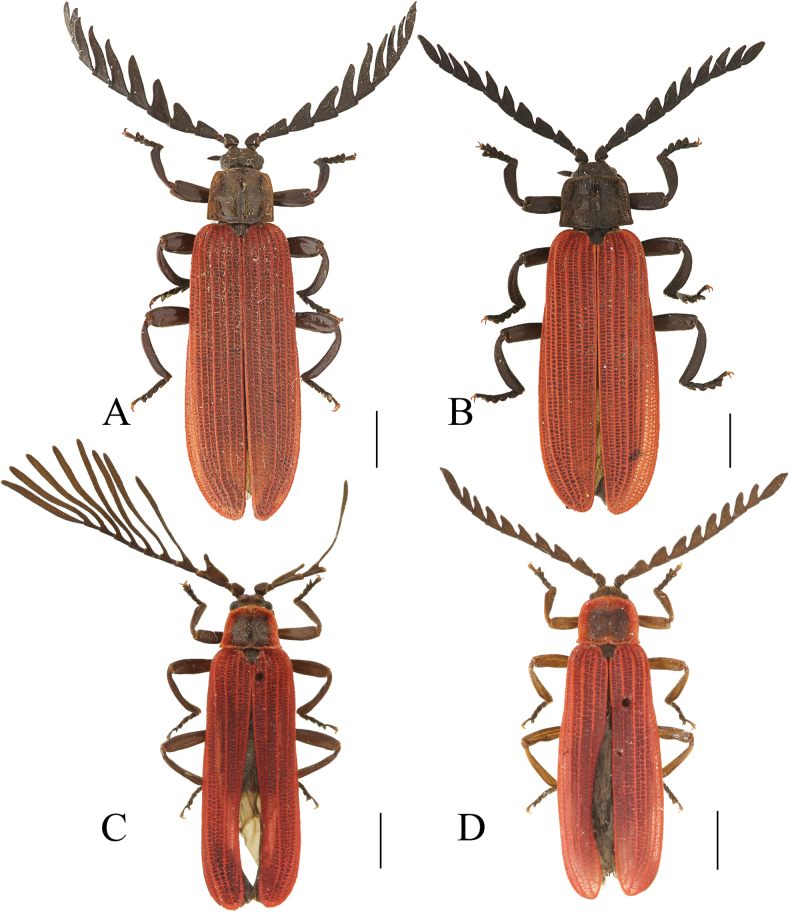
Habitus, dorsal view: *Ponyalisalternata* (Pic, 1927) (**A, B**); *P.fukiensis* (Bocak, 1999) (**C, D**). **A, C** males **B, D** females. Scale bars: 2.0 mm.

**Figure 3.**
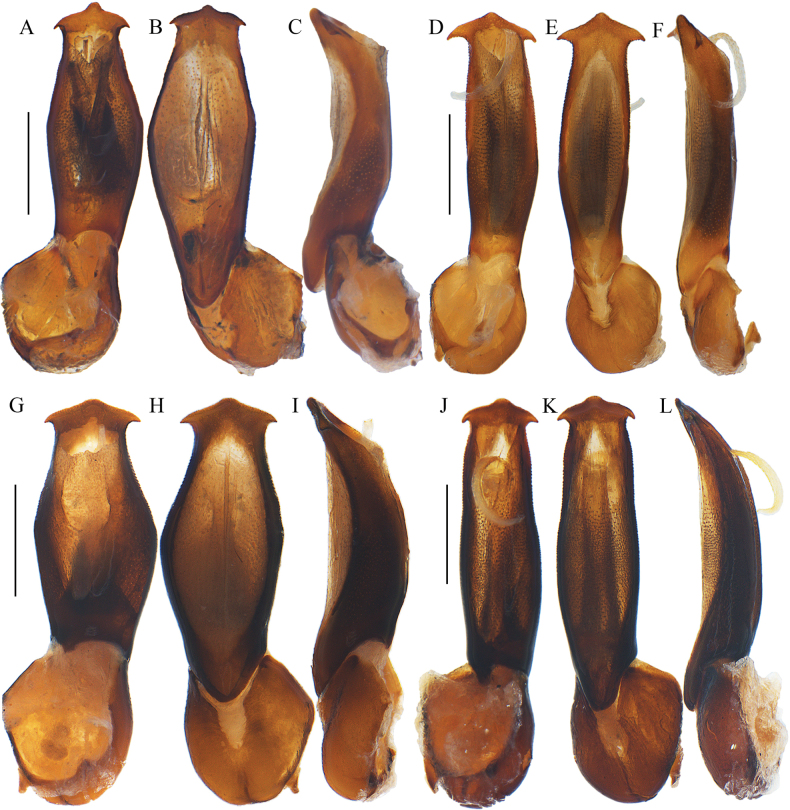
Aedeagi of *Ponyalisalternata* (Pic, 1927) (**A–C**); *P.fukiensis* (Bocak, 1999) (**D–F**); *P.himalejica* (Bourgeois, 1885) (**G–I**); *P.gracilis* (Bocak, 1999) (**J–L**). **A, D, G, J** ventral views **B, E, H, K** dorsal views **C, F, I, L** lateral views. Scale bars: 0.5 mm.

Figs 1 , 2A, B , 3A–C

Lyponia alternata Pic, 1927: 5; Bocak 1999: 96, figs 41, 78.
Ponyalis alternata : Kazantsev 2002: 205; Li et al. 2015a: 16.

#### Material examined.

China: 2♂1♀ (MHBU), Guangxi, Wuming, Damingshan, 20.V.2011, 1100 m, leg. H. Y. Liu.

#### Differential diagnosis.

This species can be readily identified from all other *Ponyalis* by the combination of the following characters: pronotum uniformly black and elytra red (Fig. 2A, B); male antennomere I flattened dorsally, III long-triangular, IV wide-triangular, lamellae of V–X extended along whole length of corresponding stem and tapered laterally, lamella of VI short and 1.3× longer than joint itself (Fig. 2A); elytral primary costae much stouter than the secondary ones in whole length, cells transverse (Fig. 2A, B); phallus widened at middle part, projected distad at apical margin and narrowly rounded at apex in ventral and dorsal views (Fig. 3A, B).

#### Descriptive notes.

**Male** (Fig. 2A). Antennae reaching elytral mid-length when inclined, antennomere I flattened dorsally, III long-triangular, 1.3× as long as wide, IV wide-triangular, approximately as long as wide, lamellae of V–X extended along whole length of corresponding stem and tapered laterally, 1.1–2.9× longer than the corresponding antennomere itself, XI fusiform and 4.2× as long as wide.

Aedeagus: phallus stout, 2.4× as long as wide, moderately widened at middle part and arcuate at lateral margins, moderately projected distad at apical margin and narrowly rounded at apex in dorsal and ventral views, with acute latero-apical angels, between which the distance much smaller than maximal width of trunk (Fig. 3A, B); almost even in width and weakly bent dorsally, truncate at ventro-apical 1/4 in lateral view (Fig. 3C).

**Female** (Fig. 2B). Similar to male, but body stouter, antennomeres III–V all nearly triangular, lamellae of VI–X 1.1–1.8× as long as its corresponding antennomere itself, XI fusiform and 3.0× as long as wide.

#### Distribution

(Fig. 1). China (Guangxi), Vietnam.

#### Remarks.

Bocak (1999) provided illustrations of basal antennomeres of male antenna and ventral view of aedeagus for this species. Here we present the images of habitus of both sexes and aedeagus in ventral, dorsal and lateral views to make its morphology better known.

### ﻿ Ponyalis fukiensis

Taxon classificationAnimaliaColeopteraLycidae

(Bocak, 1999)

F043965E-3467-5E0D-AC0B-85A1324E2E88

Figs 1 , 2C, D , 3D–F

Lyponia fukiensis Bocak, 1999: 92, figs 26, 51.
Ponyalis fukiensis : Kazantsev 2002: 206; Li et al. 2015a: 17.

#### Material examined.

China: 1♂ (MHBU), Zhejiang, Longquan, Fengyangshan, 1250 m, 31.III.2007, leg. J. Cao; 1♀ (MHBU), same locality as the preceding, 18.V.2007, leg. D. D. Hu & J. F. Gao; 1♀ (MHBU), same locality as the preceding, 17.V.2007, leg. B. F. Zhou & L. Wang; 1♀ (IZAS), Fujian, Jianyang, Huangkeng, Aotou, 26.V.1973, leg. P. Y. Yu; 1♀ (IZAS), same locality as the preceding, 800–950 m, 5.V.1960, leg. F. J. Pu.

#### Differential diagnosis.

This species can be differentiated from all others of *Ponyalis* by the combination of the following characters: pronotum black, with red margins, elytra red (Fig. 2C, D); male antennomere I nearly globular, III with outer apical angle strongly protruding laterally, lamellae of IV–X nearly parallel-sided along the whole length, lamella of IV long and 2.0× longer than joint itself, (Fig. 2C); elytral primary costae much stouter than the secondary ones in whole length, cells transverse to squared (Fig. 2C, D); phallus moderately projected distad at apical margin, narrowly rounded at apex in dorsal and ventral views (Fig. 3D, E).

#### Descriptive notes.

**Male** (Fig. 2C). Antennae reaching apical 1/4 length of elytra when inclined, antennomere I nearly globular, III long-triangular, 1.4× as long as wide, with outer apical angle strongly protruding laterally, lamellae of IV–X abruptly extended laterally and nearly parallel-sided along the whole length, 5.5–7.0× longer than the corresponding antennomere itself, XI parallel-sided and 13.0× as long as wide.

Aedeagus: phallus slender and 3.7× as long as wide, barely widened at basal part and arcuate at lateral margins, moderately projected distad at apical margin and narrowly rounded at apex in dorsal and ventral views, with sharp latero-apical angels, between which the distance barely greater than maximal width of trunk (Fig. 3D, E), almost even in width and nearly straight, truncate at ventro-apical 1/5 in lateral view (Fig. 3F).

**Female** (Fig. 2D). Similar to male, but body stouter, antennae shorter and reaching elytral apical 1/3 length when inclined, antennomeres III–V all nearly triangular, lamellae of VI–X 1.1–1.5× as long as its corresponding antennomere itself, XI fusiform and 4.0× as long as wide.

#### Distribution

(Fig. 1). China (Zhejiang, Jiangxi, Fujian, Guangdong).

### ﻿ Ponyalis himalejica

Taxon classificationAnimaliaColeopteraLycidae

(Bourgeois, 1885)

9F1ABA5E-DE35-5D41-8327-019FA0DC2DFB

**Figure 4.**
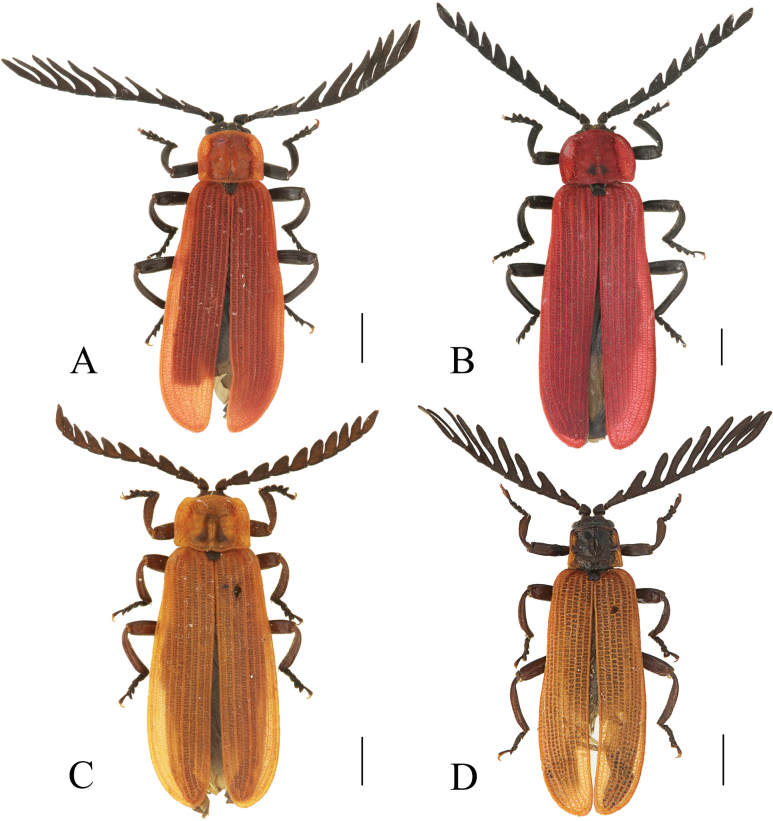
Habitus, dorsal view: *Ponyalishimalejica* (Bourgeois, 1885) (**A**–**C**); *P.gracilis* (Bocak, 1999) (**D**). **A, D** males **B, C** females. Scale bars: 2.0 mm.

Figs 1 , 3G–I , 4A–C

Lyponia himalejica Bourgeois, 1885: 79; Bocak 1999: 94, figs 52, 53.
Lyponia waterhousei Gorham, 1890: 543. Synonymized by Bocak 1999: 94.
Lyponia ochraceicollis Pic, 1923: 9. Synonymized by Bocak 1999: 94.
Lyponia aurantiaca Pic, 1927: 5. Synonymized by Bocak 1999: 94.
Lyponia robusticollis Pic, 1939: 165. Synonymized by Bocak 1999: 94.
Ponyalis himalejica : Kazantsev 2002: 205, fig. 30; Li et al. 2015a: 17.

#### Material examined.

China: 1♂1♀ (IZAS), Yunnan, Menghai, Nannuoshan, 1100–1200 m, 28.IV.1957, leg. G. J. Hong; 2♂ (IZAS), same locality as the preceding, 1600 m, 25.IV.1958, leg. G. J. Hong; 1♀ (IZAS), same locality as the preceding, 1100–1500 m, 27.IV.1957, leg. F. J. Pu; 1♀ (IZAS), Yunnan, Menghai, Chachan, 1200–1450 m, 24.IV.1957, leg. S. Y. Wang; 1♂1♀ (IZAS), Yunnan, Xishuangbanna, Mengsong, 1600 m, 26.IV.1958, leg. Y. R. Zhang; 2♂2♀ (MHBU), Yunnan, Yingjiang, Xima, 20.VII.2019, leg. P. Wang.

#### Differential diagnosis.

This species differs from all others of *Ponyalis* by the combination of the following characters: pronotum uniformly red, or present with a dark brown to black patch in center of disc, but never extending to anterior or posterior margin, elytra red (Fig. 4A–C); male antennomere I flattened dorsally, III and IV long-triangular, lamellae of V–X extended along whole length of corresponding stem and tapered laterally (Fig. 4A); elytra 5.0× longer than pronotum, primary costae nearly as strong as secondary ones, cells transverse (Fig. 4A–C); phallus strongly widened at middle part and arcuate at lateral margins, moderately projected distad at apical margin and narrowly rounded at apex in dorsal and ventral views (Fig. 3G, H).

#### Descriptive notes.

Male (Fig. 4A). Antennae reaching basal 3/5 length of elytra when inclined, antennomere I flattened dorsally, III and IV long-triangular, 1.3–1.5× as long as wide, with outer apical angles strongly protruding laterally, lamellae of V–X extended along whole length of corresponding stem and tapered laterally, 1.8–3.8× longer than the corresponding antennomere itself, XI fusiform and 5.3× as long as wide.

Aedeagus: phallus stout, 2.0× as long as wide, strongly widened at middle part and arcuate at lateral margins, moderately projected distad at apical margin and narrowly rounded at apex in dorsal and ventral views, with acute latero-apical angels, between which the distance much smaller than maximal width of trunk (Fig. 3G, H), weakly bent dorsally and tapered distad in lateral view (Fig. 3I).

**Female** (Fig. 4B, C). Similar to male, but body stouter, antennae shorter and reaching elytral mid-length when inclined, antennomeres III–V triangular, lamellae of VI–X 1.1–1.5× as long as its corresponding antennomere itself, XI fusiform and 3.0× as long as wide.

#### Distribution

(Fig. 1). China (Yunnan), Vietnam, Myanmar, Laos, Thailand, India.

#### Remarks.

We provide different habitus macrophotographs (Fig. 4B, C) for this species to show its variability of appearance, probably due to its wide distribution (Bocak 1999).

### ﻿ Ponyalis gracilis

Taxon classificationAnimaliaColeopteraLycidae

(Bocak, 1999)

5CCA3AD1-2A76-584A-9239-01F54759E58A

Figs 1 , 3J–L , 4D

Lyponia gracilis Bocak, 1999: 89, fig. 71.
Ponyalis gracilis : Kazantsev 2002: 205; Li et al. 2015a: 17.

#### Material examined.

China: 1♂ (MHBU), Fujian, Wuyishan, Guadun, 29.IV.2004, leg. D. K. Zhou.

#### Differential diagnosis.

This species can be separated from all other *Ponyalis* by the combination of the following characters: pronotum black, with red margins, elytra red (Fig. 4D); antennomere I flattened dorsally, III long-triangular, lamellae of IV–X nearly parallel-sided (Fig. 4D); elytra 5.5× longer than pronotum, primary costae as strong as the secondary ones, cells transverse (Fig. 4D); phallus bisinuate at lateral margins and narrowly rounded at apex in dorsal and ventral views (Fig. 3J, K).

#### Descriptive notes.

Male (Fig. 4D). Antennae reaching elytral mid-length when inclined, antennomere I flattened dorsally, III long-triangular, 1.5× as long as wide, lamellae of IV–X nearly parallel-sided, 1.5–3.6× longer than corresponding antennomere itself, XI parallel-sided and 8.0× as long as wide.

Aedeagus: phallus slender and 3.3× as long as wide, barely widened at basal part and bisinuate at lateral margins, moderately projected distad at apical margin and narrowly rounded at apex in dorsal and ventral views, with acute latero-apical angels, between which the distance barely smaller than maximal width of trunk (Fig. 3J, K), moderately bent dorsally and tapered distad in lateral view (Fig. 3L).

#### Distribution

(Fig. 1). China (Hunan, Fujian).

### ﻿ Ponyalis klapperichi

Taxon classificationAnimaliaColeopteraLycidae

(Bocak, 1999)

174E089F-4C64-5531-907F-11CD882E97E9

**Figure 5.**
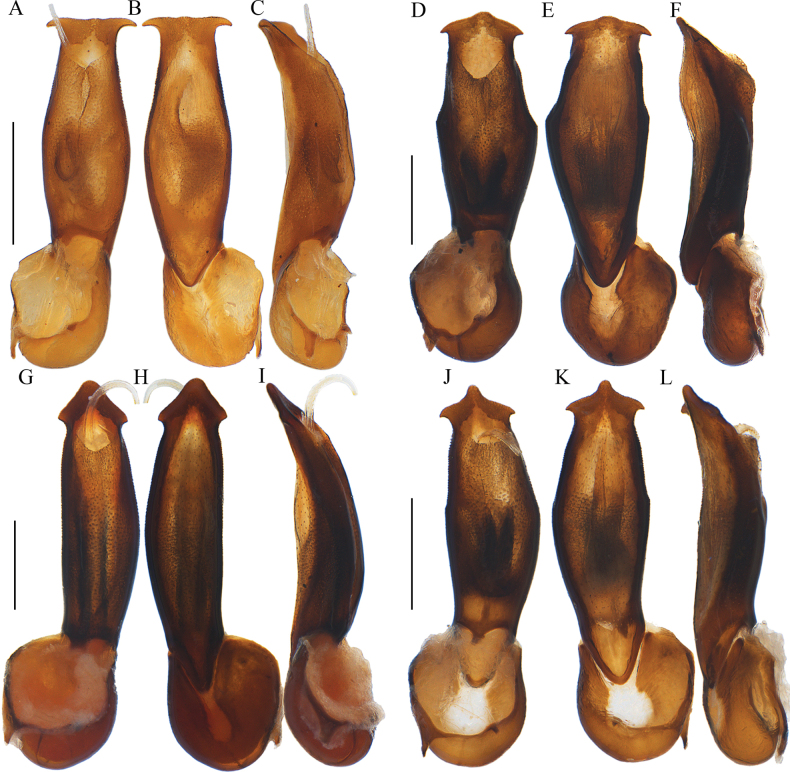
Aedeagi of *Ponyalisklapperichi* (Bocak, 1999) (**A–C**); *P.laticornis* Fairmaire, 1899 (**D–F**); *P.nigrohumeralis* (Pic, 1939) (**G–I**); *P.variabilis* Li, Bocak & Pang, 2015 (**J–L**). **A, D, G, J** ventral views **B, E, H, K** dorsal views **C, F, I, L** lateral views. Scale bars: 0.5 mm.

**Figure 6.**
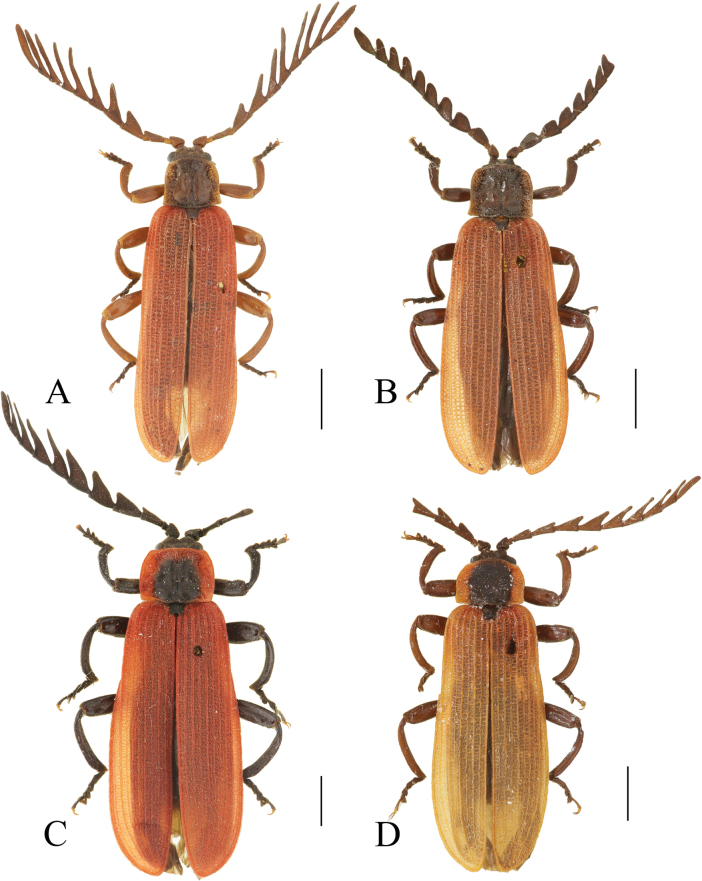
Habitus, dorsal view: *Ponyalisklapperichi* (Bocak, 1999) (**A, B**); *P.laticornis* Fairmaire, 1899 (**C, D**). **A, C, D** males **B** female. Scale bars: 2.0 mm.

Figs 1 , 5A–C , 6A, B

Lyponia klapperichi Bocak, 1999: 100, fig. 77.
Ponyalis klapperichi : Kazantsev 2002: 205; Li et al. 2015a: 17.

#### Material examined.

China: 1♂ (IZAS), Fujian, Jianyang, Huangkeng, Aotou, 850–950 m, 29.IV.1960, leg. F. J. Pu; 1♀ (IZAS), Fujian, Chongan, Xingcun, Sangang, 720 m, 16.V.1960, leg. F. J. Pu.

#### Differential diagnosis.

This species can be easily identified from the rest of the *Ponyalis* species by the combination of the following characters: pronotum uniformly black and elytra red (Fig. 6A, B); male antennomere I flattened dorsally, III long-triangular, IV wide-triangular, lamellae of VI–X nearly parallel-sided along the whole length, lamella of VI 2× longer than joint itself (Fig. 6A); elytral primary costae as strong as the secondary ones, cells transverse (Fig. 6A, B); phallus moderately widened at middle part and nearly straight at apical margin in dorsal and ventral views (Fig. 5A, B).

#### Descriptive notes.

**Male** (Fig. 6A). Antennae reaching apical 1/4 length of elytra when inclined, antennomere I nearly globular, III long-triangular, 1.4× as long as wide, IV wide-triangular and nearly as long as wide, lamella of V extended along whole length of stem and tapered laterally, lamellae of VI–X nearly parallel-sided along the whole length, 2.1–4.1× longer than the corresponding antennomere itself, XI nearly parallel-sided and 6.7× as long as wide.

Aedeagus: phallus slender and 3.7× as long as wide, moderately widened at middle part and arcuate at lateral margins, nearly straight at apical margin in dorsal and ventral views, with sharp latero-apical angels, between which the distance barely smaller than maximal width of trunk (Fig. 5A, B), almost even in width and weakly bent dorsally, truncate at ventro-apical 1/4 in lateral view (Fig. 5C).

**Female** (Fig. 6B). Similar to male, but body stouter, antennae shorter and reaching elytral mid-length when inclined, antennomeres III–V all nearly triangular, lamellae of VI–X 1.1–1.5× longer than the corresponding antennomere itself, XI fusiform and 3.2× as long as wide.

#### Distribution

(Fig. 1). China (Zhejiang, Jiangxi, Fujian), Korea.

### ﻿ Ponyalis laticornis

Taxon classificationAnimaliaColeopteraLycidae

Fairmaire, 1899

3600E1B4-9072-5332-87C8-C6B386A25869

Figs 1 , 5D–F , 6C, D

Ponyalis laticornis Fairmaire, 1899: 623; Kazantsev 2002: 205; Li et al. 2015a: 17.
Lyponia robusta Pic, 1922: 13. Synonymized by Bocak and Bocakova 2000: 42.
Lyponia laticornis : Pic, 1926: 69; Bocak 1999: 93, fig. 55.
Lyponia diversicornis Pic, 1926: 70. Synonymized by Bocak and Bocakova 2000: 42.
Lyponia limbaticollis Pic, 1926: 70. Synonymized by Bocak 1999: 93.
Lyponia guerryi Pic, 1939: 165. Synonymized by Bocak and Bocakova 2000: 42.
Lyponia patruelis Kleine, 1939: 17. Synonymized by Bocak 1999: 93.

#### Material examined.

China: 1♂ (IZAS), Yunnan, Lijiang, 3100 m, 27.V.1980, leg. L. Y. Wang; 1♂ (IZAS), Yunnan, Mancheng, 1700 m, 16.IV.1980, leg. P. Gao.

#### Differential diagnosis.

This species can be differentiated from all other *Ponyalis* by the combination of the following characters: pronotum red, present with a large black patch, extending to anterior and posterior margins, elytra red (Fig. 6C, D); male antennomere I flattened dorsally, III–IV long-triangular, lamellae of V–X extended along whole length of corresponding stem and tapered laterally (Fig. 6C); elytra 5.5× longer than pronotum and 1.2–1.3× wider than posterior margin of pronotum, primary costae nearly as strong as the secondary ones, cells transverse (Fig. 6C, D); phallus abruptly widened near middle part, moderately projected distad at apical margin and narrowly rounded at apex in dorsal and ventral views (Fig. 5D, E).

#### Descriptive notes.

**Male** (Fig. 6C, D). Antennae reaching apical 1/4 length of elytra when inclined, antennomere I flattened dorsally, III and IV long-triangular, 1.3–1.4× as long as wide, lamellae of V–X extended along whole length of corresponding stem and tapered laterally, 1.2–2.2× longer than the corresponding antennomere itself, XI fusiform and 5.0× as long as wide.

Aedeagus: phallus stout, 2.3× as long as wide, abruptly widened near middle part and obtusely angled at lateral margins, moderately projected distad at apical margin and narrowly rounded at apex in dorsal and ventral views, with acute latero-apical angels, between which the distance much smaller than maximal width of trunk (Fig. 5D, E), almost even in width and weakly bent dorsally, truncate at ventro-apical 1/4 in lateral view (Fig. 5F).

#### Distribution

(Fig. 1). China (Yunnan), Vietnam, Myanmar.

#### Remarks.

As noted by others (e.g., Bocak 1999), this species shows some variations in the coloration of pronotum, of which the black patch could be extending to anterior margin (Fig. 6D) or not (Fig. 6C).

### ﻿ Ponyalis nigrohumeralis

Taxon classificationAnimaliaColeopteraLycidae

(Pic, 1939)

88910544-6805-5F6C-9C74-09021F85C3A0

**Figure 7.**
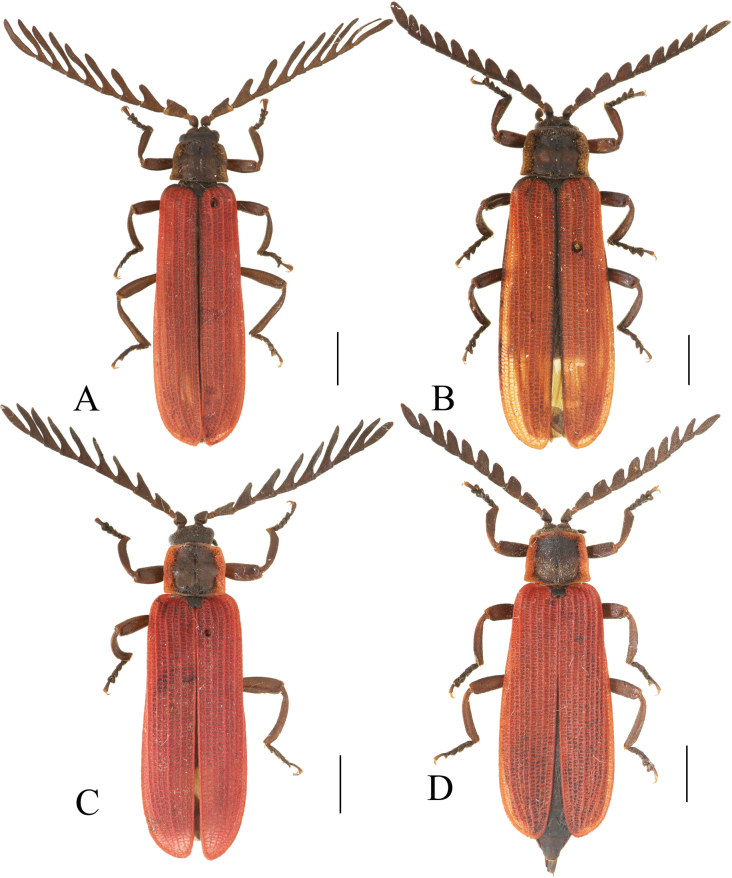
Habitus, dorsal view: *Ponyalisnigrohumeralis* (Pic, 1939) (**A, B**); *P.variabilis* Li, Bocak & Pang, 2015 (**C, D**). **A, C** males; **B, D** females. Scale bars: 2.0 mm.

Figs 1 , 5G–I , 7A, B

Lyponia nigrohumeralis Pic, 1939: 220; Bocak 1999: 100, fig. 79.
Ponyalis nigrohumeralis : Kazantsev 2002: 199, figs 31, 32; Li et al. 2015a: 17.

#### Material examined.

China: 1♂1♀ (MHBU), Shaanxi, Liuba, Miaotaizi, 10–15.VI.2005, leg. Y. B. Ba; 1♀ (MHBU), Shaanxi, Liuba, 10–12.VI.2005, Y. B. Ba leg., 1♂ (IZAS), Shaanxi, Ningshan, Pingheliang, 2106–2448 m, 1.V.2007, leg. M. Y. Lin; 1♂ (IZAS), same locality as the preceding, 1.V.2007, leg. J. Z. Cui; 2♂ (IZAS), Shaanxi, Zhouzhi, Houzhenzi, 1745–2021 m, 26.V.2007, leg. J. Z. Cui; 1♀ (IZAS), same locality as the preceding, 26.V.2007, leg. H. L. Shi; 1♂ (IZAS), Henan, Huixian, Baligou, 9–12.V.2002, leg. Y. F. Hao; 1♂ (MHBU), Gansu, Qinzhou, Niangniangba, 30.V.2021, leg. R. Liu; 1♂ (MHBU), Gansu, Wenxian, Huangtuling, 2250 m, 9.VII.2003, leg. Y. B. Ba & Y. P. Niu; 1♂ (MHBU), Sichuan, Jiuzhaigou, Wujiao, 15.VII.2009, leg. Z. H. Gao & Y. P. Niu.

#### Differential diagnosis.

This species can be easily separated from all other *Ponyalis* by the combination of the following characters: pronotum uniformly black, elytra bicolored, at least black at humeri (Fig. 7A, B); male antennomere I flattened dorsally, III long-triangular, lamellae of IV–X nearly parallel-sided along the whole length (Fig. 7A); elytral primary costae much stouter than the secondary ones in whole length, cells transverse (Fig. 7A, B); phallus moderately widened at middle part and arched at apex in dorsal and ventral views(Fig. 5G, H), weakly bent dorsally in lateral view (Fig. 5I).

#### Descriptive notes.

**Male** (Fig. 7A). Antennae reaching basal 3/5 length of elytra when inclined, antennomere I flattened dorsally, III long-triangular, 1.4× as long as wide, lamellae of IV–X nearly parallel-sided along the whole length, 1.5–3.9× longer than the corresponding antennomere itself, XI parallel-sided and 4.2× as long as wide.

Aedeagus: phallus slender and 3.3× as long as wide, moderately widened at middle part and arcuate at lateral margins, strongly projected distad at apical margin and arched at apex in dorsal and ventral views, with rectangular latero-apical angels, between which the distance barely smaller than maximal width of trunk (Fig. 5G, H), weakly bent dorsally and tapered distad in lateral view (Fig. 5I).

**Female** (Fig. 7B). Similar to male, but body stouter, antennae shorter and reaching elytral mid-length when inclined, antennomeres III–V all nearly triangular, lamellae of VI–X 1.1–1.5× as long as its corresponding antennomere itself, XI fusiform and 2.6× as long as wide.

#### Distribution

(Fig. 1). China (Henan, Shaanxi, Gansu, Hunan, Hubei, Sichuan).

#### Remarks.

Bocak (1999) and Kazantsev (2002) provided the illustration of the aedeagus for this species, and here we present the habitus of male and female for the first time.

### ﻿ Ponyalis variabilis

Taxon classificationAnimaliaColeopteraLycidae

Li, Bocak & Pang, 2015

48FBBC87-2069-5EEC-AC4F-E326C164C927

Figs 1 , 5J–L , 7C, D

Ponyalis variabilis Li, Bocak & Pang, 2015: 14, figs 8, 15, 16.

#### Material examined.

China: 1♂ (MHBU), Hubei, Qingtianpao, 22.V.2019, leg. P. Wang; 1♀ (MHBU), same locality as the preceding, 11.VI.2018, leg. P. Wang.

#### Differential diagnosis.

This species can be readily identified from all other *Ponyalis* by the combination of the following characters: pronotum black, red margins, elytra red (Fig. 7C, D); male antennomere I flattened dorsally, III long-triangular, lamellae of IV–X extended along whole length of corresponding stem and tapered laterally (Fig. 7C); elytra 5.0× longer than pronotum, primary costae much stouter than the secondary ones in whole length, cells transverse (Fig. 7C, D); phallus widened at middle part and moderately projected distad at apical margin and < 30° angle at apex in ventral and dorsal views (Fig. 5J, K).

#### Descriptive notes.

**Male** (Fig. 7C). Antennae reaching basal 3/5 length of elytra when inclined, antennomere I flattened dorsally, III long-triangular, 1.3× as long as wide, with outer apical angle barely protruding laterally, lamellae of IV–X extended along whole length of corresponding stem and tapered laterally, 2.0–3.2× longer than the corresponding antennomere itself, XI fusiform and 5.0× as long as wide.

Aedeagus: phallus slender and 3.7× as long as wide, strongly widened at middle part and arcuate at lateral margins, moderately projected distad at apical margin and narrowly narrowed at apex, with acute latero-apical angels, between which the distance much smaller than maximal width of trunk (Fig. 5J, K), almost even in width and weakly bent dorsally, truncate at ventro-apical 1/4 in lateral view (Fig. 5L).

**Female** (Fig. 7D). Similar to male, but body stouter, antennae shorter and reaching elytral mid-length when inclined, antennomeres III–V all nearly triangular, lamellae of VI–IX 1.1–1.7× as long as its corresponding antennomere itself, XI fusiform and 3.0× as long as wide.

#### Distribution

(Fig. 1). China (Hunan, Hubei, Guizhou).

### ﻿ Ponyalis dabieshanensis

Taxon classificationAnimaliaColeopteraLycidae

Y. Yang, Fang & Liu sp. nov.

2569CD85-30DD-5D79-B912-1AAB8B57176B

https://zoobank.org/35305630-EE04-48A4-9E34-7CAC75ABB912

**Figure 8.**
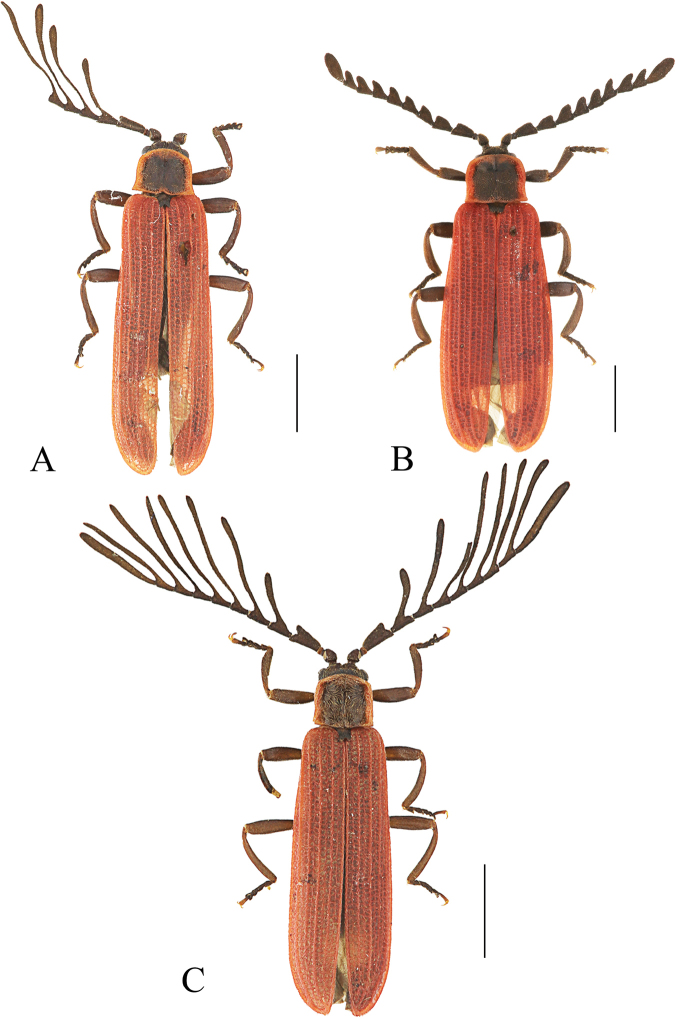
Habitus, dorsal view: *Ponyalisdabieshanensis* sp. nov. (**A, B**); *P.truncata* sp. nov. (**C**). **A, C** males **B** female. Scale bars: 2.0 mm.

**Figure 9.**
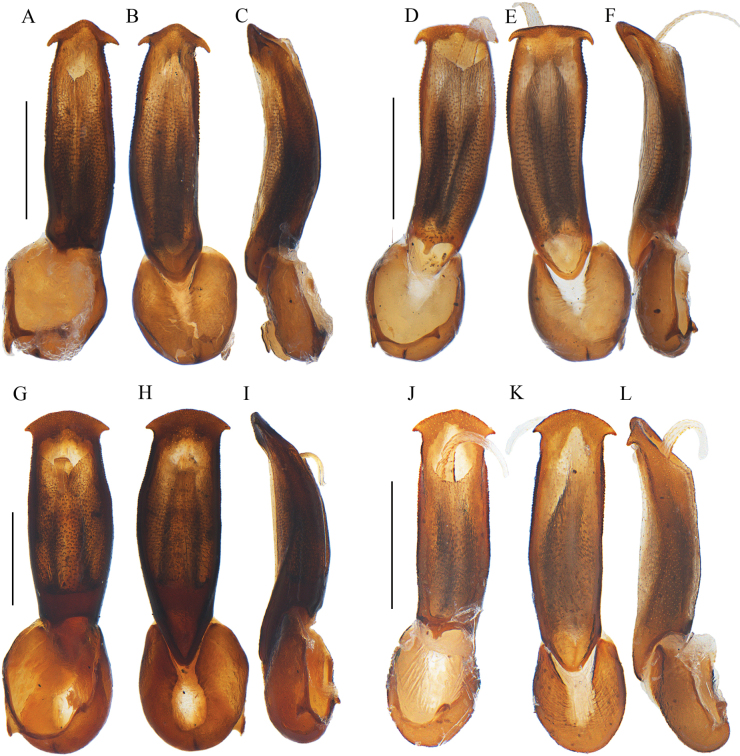
Aedeagi of *Ponyalisdabieshanensis* sp. nov. (**A–C**); *P.truncata* sp. nov. (**D–F**); *P.quadricollis* (Kiesenwetter, 1874) (**G–I**); *P.quadricollimima* sp. nov. (**J–L**). **A, D, G, J** ventral views **B, E, H, K** dorsal views **C, F, I, L** lateral views. Scale bars: 0.5 mm.

Figs 1 , 8A, B , 9A–C

#### Type material.

***Holotype***: ♂ (MHBU), China, Anhui, Yaoluoping Natural Reserve, VII. 2015, leg. J. Fang. ***Paratype***: China: 1♀ (IZAS), Anhui, Jinzhai, Baojia, Jingangtai, 5.V.2021, leg. K. D. Zhao & X. C. Zhu.

#### Differential diagnosis.

This species differs from all others of *Ponyalis* by the combination of the following characters: pronotum black, with red margins, elytra red (Fig. 8A, B); male antennomere I nearly globular, III with outer apical angle strongly protruding laterally, lamella of IV extending from middle of the joint, extremely long and 2.0× longer than joint itself (Fig. 8); elytral primary costae much stouter than the secondary ones in whole length, cells mostly squared (Fig. 8A, B); phallus moderately projected distad at apical margin and arched at apex in dorsal and ventral views (Fig. 9A, B).

The new species looks like *P.fukiensis* in the body coloration, but differs from it in the following characters: male antennomere III with outer apical angle barely protruding laterally (Fig. 8A), while strongly protruding laterally in *P.fukiensis* (Fig. 2C); phallus arched at apex in dorsal and ventral views (Fig. 9A, B), while narrowly rounded at apex in *P.fukiensis* (Fig. 3D, E); phallus with distance between the latero-apical thorns barely smaller than maximal width of trunk (Fig. 9A, B), while greater in *P.fukiensis* (Fig. 3D, E).

#### Description.

**Male** (Fig. 8A). Body stout, black to dark brown, pronotum red, with a large black patch in center of disc, elytra red.

Head dorsally flat, antennomere I nearly globular, III long-triangular, 1.4× as long as wide, with outer apical angle barely protruding laterally, lamellae of IV–VII abruptly extended laterally and nearly parallel-sided along the whole length, 4.2–7.2× longer than the corresponding antennomere itself.

Pronotum nearly trapezoidal, flat, and barely wider than long, with rounded anterior angles and acute posterior angles, anterior margin arched, lateral margins weakly sinuate and posterior margin bisinuate. Scutellum barely narrowed posteriorly and obviously emarginate at apex.

Elytra parallel-sided, all primary costae stouter than secondary ones, and primary costae II and IV stouter than other costae in whole length of elytra, most cells rectangular.

Aedeagus: phallus slender and 3.2× as long as wide, barely widened at basal part and arcuate at lateral margins, moderately projected distad at apical margin and arched at apex in dorsal and ventral views, with sharp latero-apical angels, between which the distance barely smaller than maximal width of trunk (Fig. 9A, B), almost even in width and weakly bent dorsally, truncate at ventro-apical 1/5 in lateral view (Fig. 9C).

**Female** (Fig. 8B). Similar to male, but body stouter, antennae reaching apical 1/3 length of elytra when inclined, antennomeres III–VII triangular, 1.0–1.3× as long as wide, lamellae of VIII–X 1.1–1.3× as long as its corresponding antennomere itself, XI fusiform and 3.0× as long as wide.

#### Distribution

(Fig. 1). China (Anhui).

#### Etymology.

The specific name is derived from the type locality of this new species, Dabieshan, Anhui Province, China.

#### Remarks.

The left proleg, left VIII–XI and right III–XI antennomeres of the holotype are missing.

### ﻿ Ponyalis truncata

Taxon classificationAnimaliaColeopteraLycidae

Y. Yang, Liu & X. Yang sp. nov.

02A31F7C-49B9-5206-87B9-07DF6D0811E2

https://zoobank.org/2CBD74FF-EA53-478D-8D81-56A0D8B1BEA4

Figs 1 , 8C , 9D–F

#### Type material.

***Holotype***: ♂ (IZAS), China, Anhui, Huoshan, Mozitang, Huangnibao, 902 m, 14.V.2021, leg. K. D. Zhao & X. C. Zhu. ***Paratype***: China: 1♂ (CAU), Zhejiang, Xitianmushan, V.1960, leg. J. K. Yang.

#### Differential diagnosis.

This species can be separated from all other *Ponyalis* by the combination of the following characters: pronotum black, with red margins, elytra red (Fig. 8C); male antennomere I nearly globular, III long-triangular, lamellae of IV–X abruptly extended laterally and nearly parallel-sided along the whole length, lamella of IV long and 2× longer than joint itself (Fig. 8C); elytral primary costae much stouter than the secondary ones in whole length, cells most squared (Fig. 8C); phallus widened at apical part and arcuate at lateral margins, nearly straight at apical margin in dorsal and ventral views (Fig. 9D, E).

The new species is similar to *P.fukiensis* in the body coloration and extremely long lamellae of antennomeres IV–X, but can be distinguished from the latter by the following characters: male antennomere III with outer apical angle barely protruding laterally (Fig. 8C), while strongly protruding laterally in *P.fukiensis* (Fig. 2C); phallus nearly straight at apical margin (Fig. 9D, E), while moderately projected distad in *P.fukiensis* (Fig. 3D, E); phallus with distance between the latero-apical thorns barely greater than maximal width of trunk (Fig. 9D, E), while barely smaller in *P.fukiensis* (Fig. 3D, E).

#### Description.

**Male** (Fig. 8C). Body slender, black to dark brown, pronotum red, with a black patch in the middle of disc, elytra red.

Head dorsally flat, antennae reaching apical 1/5 length of elytra when inclined, antennomere I nearly globular, III long-triangular, 1.6× as long as wide, with outer apical angle barely protruding laterally, lamellae of IV–X abruptly extended laterally and nearly parallel-sided along the whole length, 2.9–7.0× longer than the corresponding antennomere itself, XI nearly parallel-sided and 11.0× as long as wide.

Pronotum nearly trapezoidal, flat, and barely wider than long, with rounded anterior angles and acute posterior angles, anterior margin arched, lateral margins nearly straight and posterior margin bisinuate. Scutellum barely narrowed posteriorly and obviously emarginate at apex.

Elytra barely widened posteriorly, primary costae stouter than secondary ones, and primary costae II and III stouter than others in whole length of elytra, most cells squared to rectangular.

Aedeagus: phallus stout, 2.8× as long as wide, moderately widened at apical part and arcuate at lateral margins, nearly straight at apical margin in dorsal and ventral views, with sharp latero-apical thorns, between which the distance barely smaller than maximal width of trunk (Fig. 9D, E), almost even in width and weakly bent dorsally, truncate at ventro-apical 1/5 in lateral view (Fig. 9F).

**Female.** Unknown.

#### Distribution

(Fig. 1). China (Anhui, Zhejiang).

#### Etymology.

The specific name is derived from the Latin *truncatus* (cut off), referring to its phallus nearly straight at apical margin.

### ﻿ Ponyalis quadricollis

Taxon classificationAnimaliaColeopteraLycidae

(Kiesenwetter, 1874)

A5ACC366-BB42-514E-9F41-A90F98856A49

Figs 1 , 9G–I , 10A

Celetes quadricollis Kiesenwetter, 1874: 252.
Eros militans Kiesenwetter, 1874: 253. Synonymized by Lewis 1879: 16.
Lyponia quadricollis : Gorham 1883: 404; Bocak 1999: 99: figs 46, 91, 92.
Ponyalis quadricollis : Kazantsev 2002: 199; Li et al. 2015a: 17.

#### Material examined.

Japan: 1♂ (IZAS), Japan, Kyoto, 30.V.1932, leg. S. Yie.

#### Differential diagnosis.

**Figure 10.**
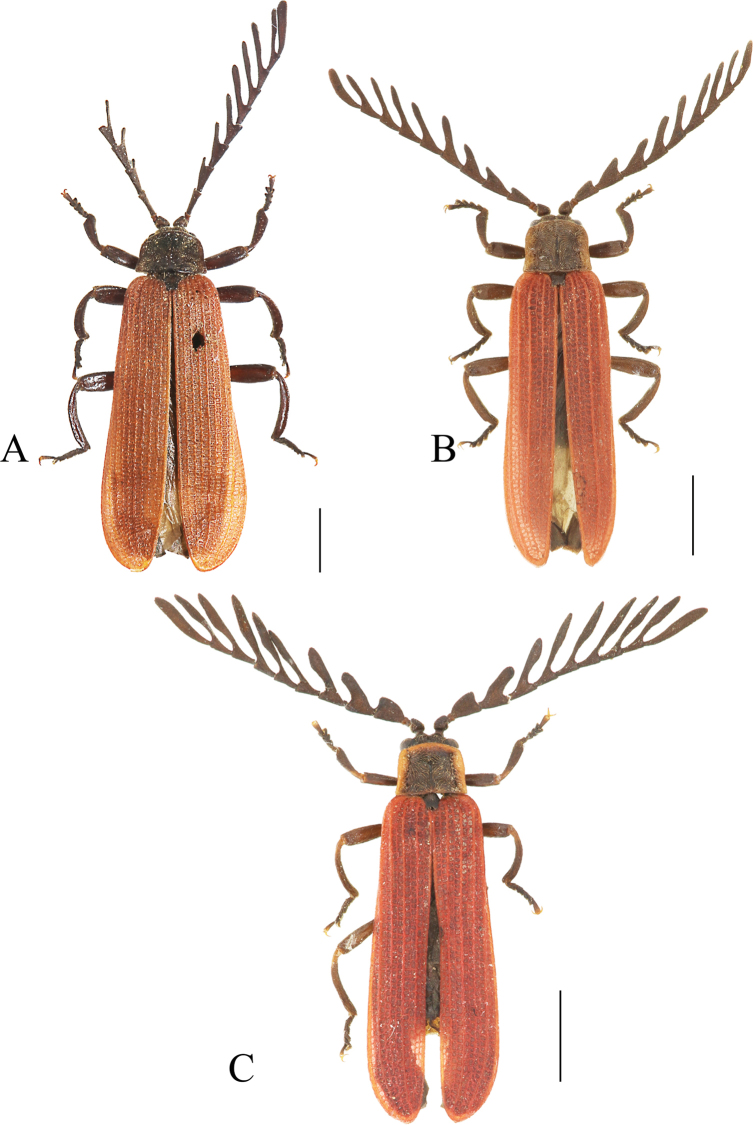
Habitus, dorsal view: *Ponyalisquadricollis* (Kiesenwetter, 1874) (**A**); *P.quadricollimima* sp. nov. (**B**); *P.longicornis* sp. nov. (**C**). **A–C** males. Scale bars: 2.0 mm.

This species can be differentiated from all other *Ponyalis* by the combination of the following characters: pronotum uniformly black, elytra red (Fig. 10A); male antennomere I flattened dorsally, III long-triangular, IV and V wide-triangular, lamellae of VI–X nearly parallel-sided along the whole length, lamella of VI longer, 1.8× longer than joint itself (Fig. 10A); elytral primary costae barely stouter than the secondary ones, cells transverse (Fig. 10A); phallus widened at middle part, moderately projected distad at apical margin and arched at apex in dorsal and ventral views (Fig. 9G, H).

#### Descriptive notes.

**Male** (Fig. 10A). Antennae reaching apical 1/5 length of elytra when inclined, antennomere I flattened dorsally, III long-triangular, 1.2× as long as wide, IV and V widely triangular, approximately as long as wide, with outer apical angels strongly protruding laterally, lamellae of VI–X nearly parallel-sided along the whole length, 2.0–3.4× longer than the corresponding antennomere itself, XI nearly parallel-sided and 5.1× as long as wide.

Aedeagus: phallus slender and 2.5× as long as wide, moderately widened at middle part and arcuate at lateral margins, moderately projected distad at apical margin and arched at apex, with acute latero-apical angels, between which the distance barely smaller than maximal width of trunk (Fig. 9G, H), weakly bent dorsally and tapered distad in lateral view (Fig. 9I).

#### Distribution

(Fig. 1). Japan.

#### Remarks.

Bocak (1999) provided male antennae and aedeagus illustrations for this species, and we present the male habitus for the first time.

### ﻿ Ponyalis quadricollimima

Taxon classificationAnimaliaColeopteraLycidae

Y. Yang, Fang & Liu sp. nov.

B05706D7-97D1-5DD9-AFE9-0B90792C09EA

https://zoobank.org/C0794C56-8095-487F-8C66-B52C7C97CD40

Figs 1 , 9J–L , 10B

#### Type material.

***Holotype***: ♂ (MHBU), China, Chongqing, Wuxi, Shuangyang, Yingtiaoling Natural Reserve, Linkouzi, 1224 m, 22.VI.2022, leg. L. Y. Wang.

#### Differential diagnosis.

The new species can be separated from all other *Ponyalis* by the combination of the following characters: pronotum uniformly black, elytral red (Fig. 10B); male antennomere I flattened dorsally, III and IV long-triangular, lamellae of V–X nearly parallel-sided along the whole length, lamella of VI longer, 1.8× longer than joint itself (Fig. 10B); primary costae much stouter than the secondary ones, cells most squared (Fig. 10B); phallus projected distad at apical margin and arched at apex in dorsal and ventral views (Fig. 9J, K).

It is most close to *P.quadricollis* in general appearance, but can be distinguished from the latter by the following characters: primary costae strongly stouter than secondary ones (Fig. 10B), while barely stouter in *P.quadricollis* (Fig. 10A); phallus barely widened at basal part in dorsal and ventral views (Fig. 9J, K), while moderately widened at middle part in *P.quadricollis* (Fig. 9G, H); phallus with distance between the latero-apical thorns barely greater than maximal width of trunk (Fig. 9J, K), while barely smaller in *P.quadricollis* (Fig. 9G, H).

#### Description.

**Male** (Fig. 10B). Body slender, black to dark brown, pronotum dark-brown, elytra red.

Head dorsally flat, antennae reaching apical 1/5 length of elytra when inclined, antennomere I flattened dorsally, III and IV long-triangular, 1.4–1.5× as long as wide, lamellae of V–X nearly parallel-sided along the whole length, 1.5–2.8× longer than the corresponding antennomere itself, XI fusiform and 5.5× as long as wide.

Pronotum trapezoidal, with rounded anterior angles and rectangular posterior angles, anterior margin arched, lateral margins sinuate and posterior margin nearly straight. Scutellum barely narrowed posteriorly and obviously emarginate at apex.

Elytra parallel-sided, all primary costae stouter than secondary ones, and primary costae I and IV stouter than others in whole length of elytra, most cells rectangular.

Aedeagus: phallus stout, 3.0× as long as wide, hardly widened at basal part, moderately projected distad at apical margin and arched at apex in dorsal and ventral views, with acute latero-apical angels, between which the distance barely greater than maximal width of trunk (Fig. 9J, K), almost even in width and nearly straight, truncate at ventro-apical 1/4 in lateral view (Fig. 9L).

**Female.** Unknown.

#### Distribution

(Fig. 1). China (Chongqing).

#### Etymology.

The name of the species is derived from the Latin *minus* (imitator), referring to its similarity to *P.quadricollis*.

### ﻿ Ponyalis longicornis

Taxon classificationAnimaliaColeopteraLycidae

Y. Yang, Liu & X. Yang sp. nov.

06D1199B-0673-5292-9869-41B2FB8D8F81

https://zoobank.org/C4B5D587-A4DD-48FB-9CC5-14650BCFB7B9

**Figure 11.**
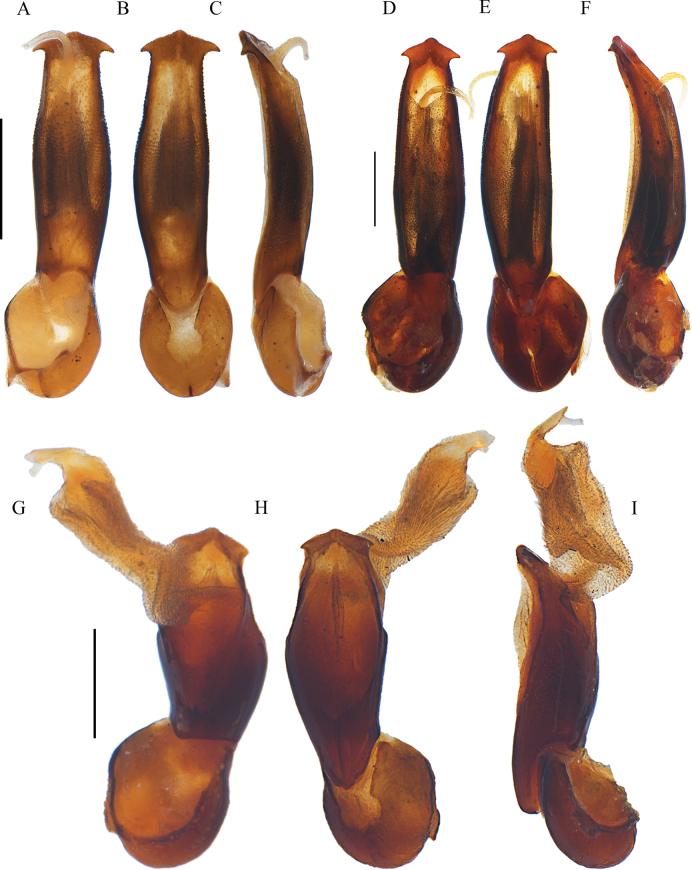
Aedeagi of *Ponyalislongicornis* sp. nov. (**A–C**); *P.zhejiangensis* sp. nov. (**D–F**); *hainanensis* sp. nov. (**G–I**). **A, D, G** ventral views **B, E, H** dorsal views **C, F, I** lateral views. Scale bars: 0.5 mm.

Figs 1 , 10C , 11A–C

#### Type material.

***Holotype***: ♂ (MHBU), China, Sichuan, Emeishan, Baoguoshi, 902 m, 29. V. 2010, leg. Q. Yuan & S. Xian.

#### Differential diagnosis.

The new species can be differentiated from the remaining *Ponyalis* species by the combination of the following characters: pronotum black, with red margins, elytra red (Fig. 10C); male antennomere I nearly globular, III long-triangular, lamellae of IV–X nearly parallel-sided along the whole length, lamella of IX 2.5× as long as joint itself (Fig. 10C); elytral primary costae much stouter than the secondary ones, cells most squared (Fig. 10C); phallus widened at middle part, moderately projected distad at apical margin and narrowly rounded at apex in dorsal and ventral views (Fig. 11A, B).

It seems similar to *P.sichuanensis* (Bocak, 1999) on basis of the general appearance, but can be easily distinguished from the latter by the following characters: lamellae of male antennomere IX 2.0× as long as joint itself (Fig. 10C), while 1.5× in *P.sichuanensis*; pronotum with a black patch extending to posterior margin (Fig. 10C), while never reaching in *P.sichuanensis*; phallus arched at apical margin (Fig. 11A, B), while nearly straight in *P.sichuanensis* (Bocak 1999: fig. 73).

#### Description.

**Male** (Fig. 10C). Body slender, black to dark brown, pronotum pale brown, with a black patch in middle of disc, which extending to posterior margin, elytra red, tibiae paler at bases.

Head dorsally flat, antennae reaching apical 1/5 length of elytra when inclined, antennomere I nearly globular, III long-triangular, 1.3× as long as wide, lamellae of IV–X nearly parallel-sided along the whole length, 2.0–3.6× longer than the corresponding antennomere itself, XI parallel-sided and 6.5× as long as wide.

Pronotum nearly trapezoidal, flat, and barely wider than long, with rounded anterior angles and rectangular posterior angles, anterior margin barely arched, lateral margins nearly straight and posterior margin nearly straight. Scutellum barely narrowed posteriorly and obviously emarginate at apex.

Elytra parallel-sided, primary costae stouter than secondary ones, and primary costae II and IV stouter than others in whole length of elytra, most cells irregular.

Aedeagus: phallus stout, 3.1× as long as wide, moderately widened at middle part and arcuate at lateral margins, moderately projected distad at apical margin and narrowly rounded at apex in dorsal and ventral views, with acute latero-apical angels, between which the distance barely smaller than maximal width of trunk (Fig. 11A, B), almost even in width and weakly bent dorsally, truncate at ventro-apical 1/5 in lateral view (Fig. 11C).

**Female.** Unknown.

#### Distribution

(Fig. 1). China (Sichuan).

#### Etymology.

The specific name is derived from the Latin *longus* (long) and *cornus* (horn), referring to its long antennae.

### ﻿ Ponyalis zhejiangensis

Taxon classificationAnimaliaColeopteraLycidae

Y. Yang, Fang & Liu sp. nov.

AF139BD3-A8F0-5EC2-8ACD-447E322DB31A

https://zoobank.org/5A418042-9D95-4DFB-8C75-F552AD1A03F1

**Figure 12.**
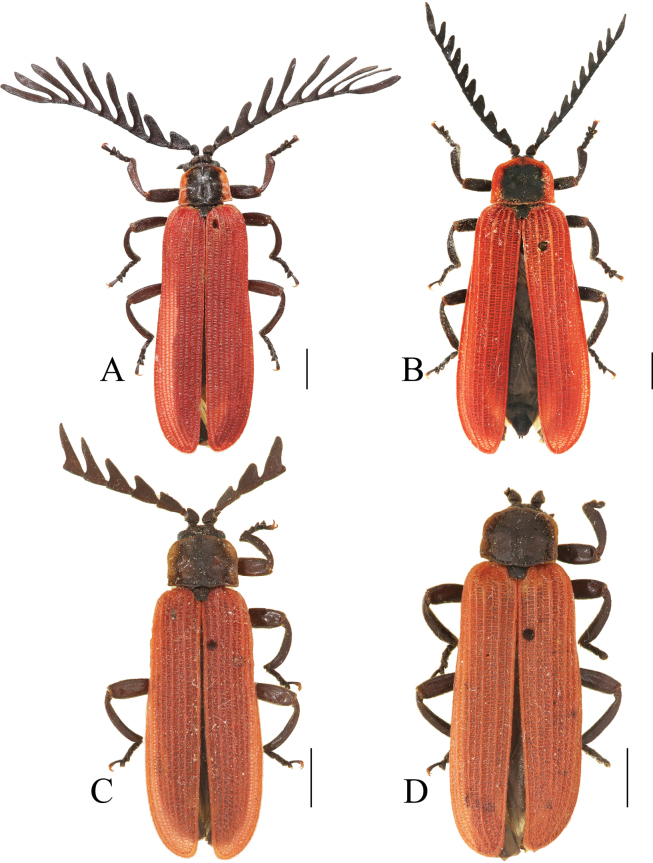
Habitus, dorsal view: *Ponyaliszhejiangensis* sp. nov. (**A, B**); *P.hainanensis* sp. nov. (**C, D**). **A, C** males **B, D** females. Scale bars: 2.0 mm.

Figs 1 , 11D–F , 12A, B

#### Type material.

***Holotype***: ♂ (MHBU), China, Zhejiang, Longquan, Fenyangshan, 1250 m, 17.V.2007, leg. B. F. Zhou & L. Wang. ***Paratype***: 1♀ (MHBU), same locality as holotype, 1500 m, 15.V.2007, leg. J. H. Xu & L. Q. Liu.

#### Differential diagnosis.

This new species can be separated from all other *Ponyalis* by the combination of the following characters: pronotum black, with red margins, elytra red (Fig. 12A, B); male antennomere I nearly globular, III long-triangular, lamellae of IV–X nearly parallel-sided along the whole length (Fig. 12A); elytra 5.5× longer than pronotum, primary costae barely stouter than the secondary ones only basally, cells transverse (Fig. 12A, B); phallus widened at basal part and narrowly rounded at apex in dorsal and ventral views (Fig. 11D, E).

It looks similar to *P.gracilis* in the coloration, but differs in the following characters: anterior margin of pronotum arched (Fig. 12A), while nearly straight in *P.gracilis* (Fig. 4D); phallus arcuate at lateral margins in dorsal and ventral views (Fig. 11D, E), while bisinuate in *P.gracilis* (Fig. 3J, K); phallus with distance between the latero-apical thorns much smaller than maximal width of trunk (Fig. 11D, E), while barely smaller in *P.gracilis* (Fig. 3J, K).

#### Description.

**Male** (Fig. 12A). Body stout, black, pronotum cinnabar red, with a black patch in middle of disc, which extending to both anterior and posterior margins, elytra red.

Head dorsally flat, antennae reaching elytral mid-length when inclined, antennomere I flattened dorsally, III long-triangular, 1.3× as long as wide, lamellae of IV–X nearly parallel-sided along the whole length, 1.7–4.7× longer than the corresponding antennomere itself, XI parallel-sided and 6.7× as long as wide.

Pronotum trapezoidal, flat, and wider than long, with rounded anterior angles and acute posterior angles, anterior margin arched, lateral margins nearly straight and posterior margin weakly bisinuate. Scutellum narrowed posteriorly and obviously emarginate at apex.

Elytra barely widened posteriorly, primary costae barely stouter than secondary ones only at the humeral part, cells squared to transverse.

Aedeagus: phallus stout, 3.3× as long as wide, moderately widened at basal part and arcuate at lateral margins, moderately projected distad at apical margin and narrowly rounded at apex in dorsal and ventral views, with acute latero-apical angels, between which the distance much smaller than maximal width of trunk (Fig. 11D, E), weakly bent dorsally and tapered distad in lateral view (Fig. 11F).

**Female** (Fig. 12B). Similar to male, but body stouter, antennae reaching basal 1/3 length of elytra when inclined, antennomeres III–V all nearly triangular, lamellae of VI–X 1.1–1.5× longer than the corresponding antennomere itself, pronotum with black patch never extending to anterior or posterior margin.

#### Distribution

(Fig. 1). China (Zhejiang).

#### Etymology.

The name of the species is derived from the name of the type locality, Zhejiang Province, China.

### ﻿ Ponyalis hainanensis

Taxon classificationAnimaliaColeopteraLycidae

Y. Yang, Liu & X. Yang sp. nov.

E52A2806-0871-51FB-8C0A-A2AB179E11C1

https://zoobank.org/55E7F4A5-01DE-4E0E-8758-5931A0EDB583

Figs 1 , 11G–I , 12C, D

#### Type material.

***Holotype***: ♂ (IZAS), China, Hainan, Jianfeng, 21.V.1980, leg. F. J. Pu. ***Paratype***: 1♀ (IZAS), same data as the holotype.

#### Differential diagnosis.

This new species can be readily identified from all other *Ponyalis* by the combination of the following characters: pronotum uniformly black, elytra red (Fig. 12C, D); male antennomere I flattened dorsally, III and IV long-triangular, lamella of VI longer, 1.8× longer than joint itself (Fig. 12C); elytral primary costae much stouter than the secondary ones, cells transverse (Fig. 12C, D); phallus widened near middle and sinuate at lateral margins, projected distad and narrowly rounded at apical margin in dorsal and ventral views (Fig. 11G, H).

It looks like *P.klapperichi* in the general appearance, but can be distinguished from the latter by the following characters: male pronotum broad (0.7× longer than wider) (Fig. 12C), while slender in *P.klapperichi* (0.9–1.0× longer than wider) (Fig. 6A); lamella of antennomere VII 2.3× longer than joint itself (Fig. 12C), while 3.0× in *P.klapperichi* (Fig. 6A); phallus moderately projected distad at apical margin (Fig. 11G, H), while nearly straight in *P.klapperichi* (Fig. 5A, B).

#### Description.

**Male** (Fig. 12C). Body stout, black, pronotum dark-brown, elytra cinnabar red.

Head dorsally flat, antennae reaching elytral mid-length when inclined, antennomere I barely flattened dorsally, III–IV long-triangular, 1.3–1.5× as long as wide, lamellae of V–VII extended along whole length of corresponding stem and tapered laterally, 1.5–2.3× longer than the corresponding antennomere itself.

Pronotum trapezoidal, flat, and barely wider than long, with rounded anterior angles and rectangular posterior angles, anterior margin arched, lateral margins weakly sinuate and posterior margin bisinuate. Scutellum barely narrowed posteriorly and obviously emarginate at apex.

Elytra barely widened posteriorly, primary costae stouter than secondary ones, primary costae II, III and IV stouter than others in whole length of elytra, most cells rectangular.

Aedeagus: phallus stout, 2.1× as long as wide, abruptly widened at middle and sinuate at lateral margins, moderately projected distad at apical margin and narrowly rounded at apex in dorsal and ventral views, with acute latero-apical angels, between which the distance much smaller than maximal width of trunk (Fig. 11G, H), almost even in width and weakly bent dorsally, truncate at ventro-apical 1/4 in lateral view (Fig. 11I); internal sac membranous and expanded, densely covered with minute tubercles and short bristles on surface, abruptly thinned into a thorn-like apex (Fig. 11 G–I).

**Female** (Fig. 12D). Similar to male, but body stouter.

#### Distribution

(Fig. 1). China (Hainan).

#### Etymology.

The name of the species is derived from the name of the type locality, Hainan Island, China.

#### Remarks.

The left pro- and meso-legs, left VIII–XI and right VI–XI antennomeres of the holotype, and both antennomeres II–XI, left proleg, and the right pro- and mesotarsomeres III–V of the paratype are missing.

## ﻿Discussion

As the number of species descriptions increases, we have a better understanding of the diversity of *Ponyalis*. With six species newly described, we raised the number of known species to 24. In general (Fig. 1), *Ponyalis* are mainly distributed in China (21 species, accounting for 87.5% of species diversity), and most of them are endemic, except for a few that are distributed to adjacent countries, including Vietnam, Myanmar, Laos, Thailand, and India (3 species, 12.5%), and Korea (1 species, 4.1%), respectively. The remaining three species (12.5%) are restricted to Japan. This distribution pattern is similar to that of *Lyponia* (Fang et al. 2024). As noted by others (Nakane 1969; Bocak 1999), there is a high turnover of Lyponiini in species composition amongst mountain ranges in China and between continental Asia and adjacent islands (including Taiwan and Japan). The tribe was inferred to have originated from continental Asia, and the species in adjacent islands were established separately by multiple vicariance or short distance dispersal events (Li et al. 2015b; Masek et al. 2018). Meanwhile, most of the *Ponyalis* species are narrowly distributed (Fig. 1), and limited ranges also have often been documented in other net-winged beetles, which have been ascribed to their low dispersal propensity (e.g., Bocak and Yagi 2010; Malohlava and Bocak 2010; Sklenarova et al. 2013). The present result is congruent with the opinion of Li et al. (2015b) that allopatric speciation is proposed as the predominant mechanism of speciation of *Ponyalis*.

Furthermore, based on the examination and comparison results of more material, we have a better understanding of the morphology of *Ponyalis*. The internal sac of male genitalia is usually invaginated in the phallus and exposed only apically, making it difficult to be well prepared and retracted for examination. Luckily, we have almost seen the overall structure of internal sac of *P.hainanensis* sp. nov. Our examination shows that the internal sac (Fig. 11G–I) is membranous and densely covered with minute tubercles and short bristles on surface, and it is overall expanded, but abruptly thinned at apex. The apex is a slender or thorn-shaped tube, and either long and evidently exposed (e.g., Figs 3D–F, J–L, 5A–C, G–L) or short even hardly visible (e.g., Figs 3A–C, G–I, 5D–F, 9, 11). However, this is inconsistent with the opinion of Li et al. (2015a), who argued that the apical length of internal sac is a differential diagnosis between *Ponyalis* and Lyponia (Poniella) Kazantsev, 2002. These have nearly identical shapes of the aedeagus (Li et al. 2015a), but their separation was well supported by the molecular phylogenetic analysis (Li et al. 2015b), also by some morphological differences found in the antennae, elytral costae, and coxite (Kazantsev 2002). This suggests that we should consider and integrate the characters comprehensively, and not base taxonomic decisions on a single character.

Moreover, Li et al. (2015b) noted that the females do not have any diagnostic characters of either the genitalia or antennae, but we found that they are indeed present with some differences in the details of their antennal shapes (e.g., Figs 2B, D, 4B, C, 6B, 7B, D, 8B, 12B). Of course, this requires the material of both sexes available for us to recognize the species comprehensively. Within *Ponyalis*, although the appearance is sometimes variable, such as *P.himalejica* and *P.laticornis*, its antennal shape and aedeagus are relatively conserved and dependable, which are mainly applied in the following identification key to the species.

## Supplementary Material

XML Treatment for Ponyalis

XML Treatment for Ponyalis alternata

XML Treatment for Ponyalis fukiensis

XML Treatment for Ponyalis himalejica

XML Treatment for Ponyalis gracilis

XML Treatment for Ponyalis klapperichi

XML Treatment for Ponyalis laticornis

XML Treatment for Ponyalis nigrohumeralis

XML Treatment for Ponyalis variabilis

XML Treatment for Ponyalis dabieshanensis

XML Treatment for Ponyalis truncata

XML Treatment for Ponyalis quadricollis

XML Treatment for Ponyalis quadricollimima

XML Treatment for Ponyalis longicornis

XML Treatment for Ponyalis zhejiangensis

XML Treatment for Ponyalis hainanensis

## ﻿Additional information

### Author ORCIDs

Chen Fang https://orcid.org/0009-0005-0483-8446

Yuxia Yang https://orcid.org/0000-0002-3118-6659

Xingke Yang https://orcid.org/0000-0003-3676-6828

Haoyu Liu https://orcid.org/0000-0003-1383-5560

### Data availability

All of the data that support the findings of this study are available in the main text.
